# The platinum coordination complex inhibits cell invasion-migration and epithelial-to-mesenchymal transition by altering the TGF-β-SMAD pathway in colorectal cancer

**DOI:** 10.3389/fphar.2023.1178190

**Published:** 2023-11-08

**Authors:** Maha-Hamadien Abdulla, Aminah Ahmad Alzailai, Mansoor-Ali Vaali-Mohammed, Rehan Ahmad, Sabiha Fatima, Ahmed Zubaidi, Thamer bin Traiki, Amer Mahmood, Reem Hamoud Alrashoudi, Zahid Khan

**Affiliations:** ^1^ Colorectal Research Chair, Department of Surgery, College of Medicine, King Saud University, Riyadh, Saudi Arabia; ^2^ Department of Clinical Laboratory Sciences, College of Applied Medical Sciences, King Saud University, Riyadh, Saudi Arabia; ^3^ Stem Cell Unit, Department of Anatomy, King Saud University College of Medicine, Riyadh, Saudi Arabia; ^4^ Genome Research Chair, Department of Biochemistry, College of Science, King Saud University, Riyadh, Saudi Arabia

**Keywords:** platinum, colorectal cancer, epithelial-to-mesenchymal transition, metastasis, TGF-β

## Abstract

**Introduction:** There is a steady increase in colorectal cancer (CRC) incidences worldwide; at diagnosis, about 20 percent of cases show metastases. The transforming growth factor-beta (TGF-β) signaling pathway is one of the critical pathways that influence the expression of cadherins allowing the epithelial-to-mesenchymal transition (EMT), which is involved in the progression of the normal colorectal epithelium to adenoma and metastatic carcinoma. The current study aimed to investigate the impact of a novel coordination complex of platinum (salicylaldiminato) PT(II) complex with dimethyl propylene linkage (PT-complex) on TGF-β and EMT markers involved in the invasion and migration of the human HT-29 and SW620 CRC cell lines.

**Methods:** Functional study and wound healing assay showed PT-complex significantly reduced cell motility and the migration and invasion of CRC cell lines compared to the untreated control. Western blot performed in the presence and absence of TGF-β demonstrated that PT-complex significantly regulated the TGF-β-mediated altered expressions of EMT markers.

**Results and Discussion:** PT-complex attenuated the migration and invasion by upregulating the protein expression of EMT-suppressing factor E-cadherin and suppressing EMT-inducing factors such as N-Cadherin and Vimentin. Moreover, PT-complex significantly suppressed the activation of SMAD3 in both CRC cell lines. Further, the microarray data analysis revealed differential expression of genes related to invasion and migration. In conclusion, besides displaying antiproliferative activity, the PT complex can decrease the metastasis of CRC cell lines by modulating TGF-β-regulated EMT markers. These findings provide new insight into TGF-β/SMAD signaling as the molecular mechanism involved in the antitumoral properties of novel PT-complex.

## Introduction

Globally, colorectal cancer (CRC) is reported as the third most common malignancy diagnosed in men and the second most common cancer in women (Sawicki et al., 2021; Siegel et al., 2023). In 2018, 881,000 deaths and 1.8 million new cases have been reported across the world (Bray et al., 2018). Metastasis occurs in 18% of patients with rectal cancer and 20%–25% of patients with colon cancer at initial diagnosis (Riihimaki et al., 2016). Epithelial-to-mesenchymal transition (EMT) is a critical step in enhancing cancer invasion and metastasis, which plays a significant role in the progression of CRC (Vu and Datta, 2017; Wu et al., 2020; Huang et al., 2022). Among various signaling pathways that trigger EMT, the TGF*-*β signaling pathway has been implicated as a primary inducer of EMT (Lu et al., 2020).

The adherens junction also plays an essential role in regulating the activity of the entire junctional complex. Cadherins are calcium-dependent cell adhesion and major adhesion junction molecules (Adil et al., 2021). Cadherins expressed by most epithelial tissues include epithelial cadherin (E-cadherin). In many epithelial-derived cancer cells, the loss of E-cadherin expression correlates with an invasive and undifferentiated phenotype (Christou et al., 2017). Another adhesion molecule is neural cadherin (N-cadherin), which is linked to enhanced invasive capacity in cancer. Thus, the “cadherin switching” due to the loss of E-cadherin expression and gain in N-cadherin expression in cancer cells has a functional impact on cancer progression (Loh et al., 2019; Kaszak et al., 2020). It has been observed that CRC tumors carrying an activated TGF-β pathway in CRC patients are associated with a poorer prognosis than those with CRC tumors that do not have TGF-β activation. Numerous approaches to inhibit the TGF-β pathway have emerged as anti-cancer therapies (Itatani et al., 2019). Currently, the effective treatment options for CRC include surgery, radiotherapy, and chemotherapy (Dagoglu et al., 2015; Gustavsson et al., 2015).

Over the past decades, various chemotherapeutic agents have improved CRC patient survival rates. However, adverse side effects, such as drug resistance, poorly targeted delivery, and death of normal cells, have limited the efficacy of these drugs due to therapeutic failure (Rejhová et al., 2018; Zhang et al., 2022). Recently, the less toxic and more stable platinum-based anticancer drug, the Schiff base (salicylaldiminato) PT complex (II) with dimethylpropylene linkage (PT-complex), has been developed to improve CRC and other cancer treatment outcomes (Azam et al., 2017). Our previous study was the first to show a dose-dependent antiproliferative potential of platinum complex (PT) on human colorectal cancer cell lines (Al-Khayal et al., 2020). Based on our previous observations, the current study intended to assess the potential of the PT complex on the invasion and migration of HT-29 and SW-620 CRC cell lines to elucidate its potential to suppress cancer metastasis. Our findings indicate that treatment with the PT complex inhibited cell invasion, migration, and EMT marker expression in both cell lines. This indicates that the coordination complex of PT may potentially block cancer metastasis.

## Materials and methods cell culture

Human CRC HT-29 (colorectal adenocarcinoma [HTB-38] cell lines and human SW620 [CCL- 227] metastatic CRC cell lines) were purchased from ATCC (Manassa, VA, United States). Cells were maintained and cultured in Roswell Park Memorial Institute (RPMI) 1640 medium containing 10% heat-inactivated fetal bovine serum (Thermo Fisher Scientific Inc., Waltham, MA, United States). The medium was supplemented with 10% heat-inactivated fetal bovine serum, 100 units/mL streptomycin, 100 units/mL penicillin, and 2 mmol/l L-glutamine, all procured from Thermo Fisher Scientific Inc., Waltham, MA, United States of America. Cells were grown at 37°C with 5% CO_2_ in a CO_2_ incubator (Thermo Scientific Forma Series II). Cells were monitored and observed using an inverted light microscope (MICROS, Sundew MCX 1600, Austria) with image software (MICROS, Austria). PT complex was obtained from the Department of Chemistry, College of Science, King Saud University (Azam et al., 2017). PT complex was dissolved in DMSO at 10 mM and stored at −20 C for further use. For this study, two concentrations of PT complex (5 Μm and 10 μM) were used.

## Wound healing assay

The HT29 and SW620 cells were seeded in duplicate at 1×10^6^ cells per well and incubated for 3 days to reach confluency. A scratch of a wound was made in the center of each well using a sterile 10-μL pipette tip under standard conditions. Detached cells were removed by gently washing three times with PBS (Bio-Rad 1610780) and replaced with 2 mL of complete culture medium. PT complex (10 μM) was added to each well, leaving one well as a control. The ability and motility of the cells to close the wound were tested by measuring the healing area. This was carried out by taking an image at 0 times (t0) of scratch under a microscope (10×) using an inverted light microscope (MICROS, Sundew MCX 1600, Austria) with the assistance of image software (MICROS, Austria). Then, plates were incubated for 48 h (t final) at 37°C in a CO_2_ incubator (Sanyo, MCO175). After final incubation, the second images were retaken, and the average gap widths at t0 and t final were measured and normalized to control cells using image software (MICROS, Austria) (Al-Khayal et al., 2017).

## Measurement of the invasion and migration of CRC cells

The concentration-dependent inhibitory effect was evaluated by measuring the PT complex (5 and 10 µM) on the invasion and migration of both cell lines (HT-29 and SW620). To track cell migration and invasive ability in real-time, the xCELLigence Real-Time Cell Analyzer DualPlate (RTCA-DP) instrument was employed as recommended by the manufacturer (Acea Biosciences Inc. USA) (Al-Khayal et al., 2017). The cell migration assays used a 16-well CIM (cellular invasion/migration) plate (CIM-16, Roche Diagnostics GmbH, Mannheim, Germany). The CIM-16 plate has two chambers: an upper and lower chamber. For CIM-Plate-16 preparation, the upper chamber of the CIM-16 plate was filled with serum-free medium to hydrate the membrane, and the lower chamber was filled with complete medium (10% FBS). Then, after the incubation period and before adding the cells, the CIM-16 plates were placed in the RTCA DP machine station, and the baseline measurement step was performed against the signal cell-free medium as the background. Then, HT-29 and SW620 cells were counted, and 12,000 cells/well were seeded into the wells of the upper chamber. Cells in CIM-16 plates were placed in the RTCA DP station, and migration was monitored every 15 min for 200 h. Cell migration is measured and represented by the changes in electrical impedance measured by the gold microelectrode plated on the underside of the membrane of the upper chamber. The increase in impedance is directly proportional to the rise in the number of cells that migrated on the underside of the membrane. Cell index values were monitored and recorded every 15 min for 200 h. The RTCA software 1.2.1 of the RTCA xCELLigence system was used to calculate the cell index values and for plotting. Then, cell index curves were monitored for 200 h to evaluate cell migration. The same protocol was applied for the cell invasion assay using the xCELLigence system and CIM-16 plate, except that the upper chamber was first coated with Matrigel (356234, BD Biosciences).

## Protein extraction

SW620 and HT-29 cells maintained in RPMI medium were treated with PT complex (5 and 10 μM) and incubated with 5% CO_2_ for 24 h at 37°C. In certain experiments, cells were treated with TGF-β (10 ng/mL) for 30 min at 37°C with 5% CO_2_ before cell harvesting to detect SMAD3 phosphorylation and for a 24-h incubation at 37°C with 5% CO_2_ for EMT markers. For total cell lysate preparation, cell pellets were incubated (15 min) with radioimmunoprecipitation assay (RIPA) buffer (RIPA buffer, Pierce, 89900, Thermo Scientific) at 4°C using ice and then centrifuged at 14,000 rpm for 15 min. Then, after the centrifugation, the supernatant was collected and was considered as the total cell lysates containing soluble proteins. The protein concentrations were measured using a Bio-Rad SmartSpec Plus spectrophotometer employing a Bradford protein assay reagent (Bio-Rad Laboratories, Hercules, CA, USA).

## Western blotting

SW620 and HT-29 cells maintained in RPMI medium were treated with PT complex (5 and 10 μM) and incubated with 5% CO_2_ for 24 h at 37°C. In certain experiments, cells were treated with TGF-β (10 ng/mL) for 30 min at 37°C with 5% CO_2_ before cell harvesting to detect SMAD3 phosphorylation and for 24 h incubation at 37°C with 5% CO_2_ for EMT markers. For total cell lysate preparation, cell pellets were incubated (15 min) with RIPA buffer (Pierce, 89900, Thermo-Scientific) at 4°C using ice and then centrifuged at 14,000 rpm for 15 min. Then, after centrifugation, the supernatant was collected and considered total cell lysates containing soluble proteins. The protein concentrations were measured on a Bio-Rad SmartSpec Plus spectrophotometer employing a Bradford protein assay reagent (Bio-Rad Laboratories, Hercules, CA, USA). Equal volumes of cell lysate proteins (20 μg) were loaded and electrophoresed using 4%–20% Mini-Protean TGX precast gels (Bio-Rad Laboratories, Hercules, CA USA) on running buffer consisting of 1× Tris/glycerin/SDS buffer at 150 V for 2 h. Subsequently, the gel was transferred into a nitrocellulose membrane using the trans-blot turbo transfer system (TransBlot Turbo transfer pack, Bio-Rad Laboratories, Hercules, CA USA) and blocked in 5% skimmed milk in PBS containing 0.1% Tween 20 (PBST) for 1 h at room temperature. Then, the blocked membranes were washed twice with PBST. After that, primary antibodies in PBST solution at a 1:1000 dilution were added to the membranes, which were then incubated overnight at 4°C. The following primary antibodies were used: anti-E-cadherin (Santa Cruz Biotechnology, sc-8426); anti-N-cadherin (Santa Cruz Biotechnology, sc-8424); Vimentin (Santa Cruz Biotechnology, sc-6260); Phospho-SMAD3 (Santa Cruz Biotechnology, sc- 517575); SMAD3 (Santa Cruz Biotechnology, sc-7960); and β-Actin, the internal control (Santa Cruz Biotechnology, sc-47778). After overnight incubation, the membranes were washed with PBST (PBS supplement with Tween-20 0.1%) two times and incubated with secondary antibodies against HRP-conjugated mouse 1:3000 (Santa Cruz Biotechnology, sc- 516102) and HRP-conjugated rabbit 1:3000 (Santa Cruz Biotechnology, sc-2357) for 1 h at room temperature. For the secondary HRP-conjugated antibodies, 0.2 gm skimmed milk powder in 10 mL PBST solution was used as a diluent. Then, membranes were washed twice with PBST and once with PBS. Finally, the membranes were incubated with chemiluminescence western HRP substrate (Immobilon Crescendo, WBLUR0500, Millipore). Chemiluminescence was detected using a blot scanner (LI-COR) and densitometry analysis using Image Studio digits Ver (LI-COR Biosciences). The intensity of the protein bands was semi-quantified relative to β-actin, which was used as an internal control.

## RNA extraction

Extraction of total RNA was carried out using a PureLink RNA mini kit procured from Ambion (Life Technologies, USA, 12183018A), and quantification was performed using a NanoDrop 2000 spectrophotometer (Thermo Scientific, Wilmington, DE, USA).

## Gene expression microarray

Extraction of total RNA was performed using a PureLink RNA mini kit, and quantification was performed using a NanoDrop 2000 spectrophotometer. After labeling the RNA, it was initially hybridized to the Agilent Human SurePrint G3 Human GE 8 × 60 k microarray chip (Agilent Technologies). Microarray analysis was performed at the King Saud University College of Medicine’s Stem Cell Unit. Normalization and data analysis were conducted using GeneSpring GX software (Agilent Technologies). Path analysis was conducted using the Single Experiment Pathway analysis feature in GeneSpring 12.0 (Agilent Technologies). A twofold cutoff with *p* < 0.02 was used.

### Statistical analysis

Results are presented as the mean (mean ± SD) of three independent experiments. Statistical analysis was conducted using GraphPad Prism7 (GraphPad Software Inc.). Differences between the control and treated groups were compared using Student’s t-test. Statistical significance was determined at a *p*-value less than 0.05.

## Results

### PT complex decreases invasion and migration in CRC cells

**FIGURE 1:**
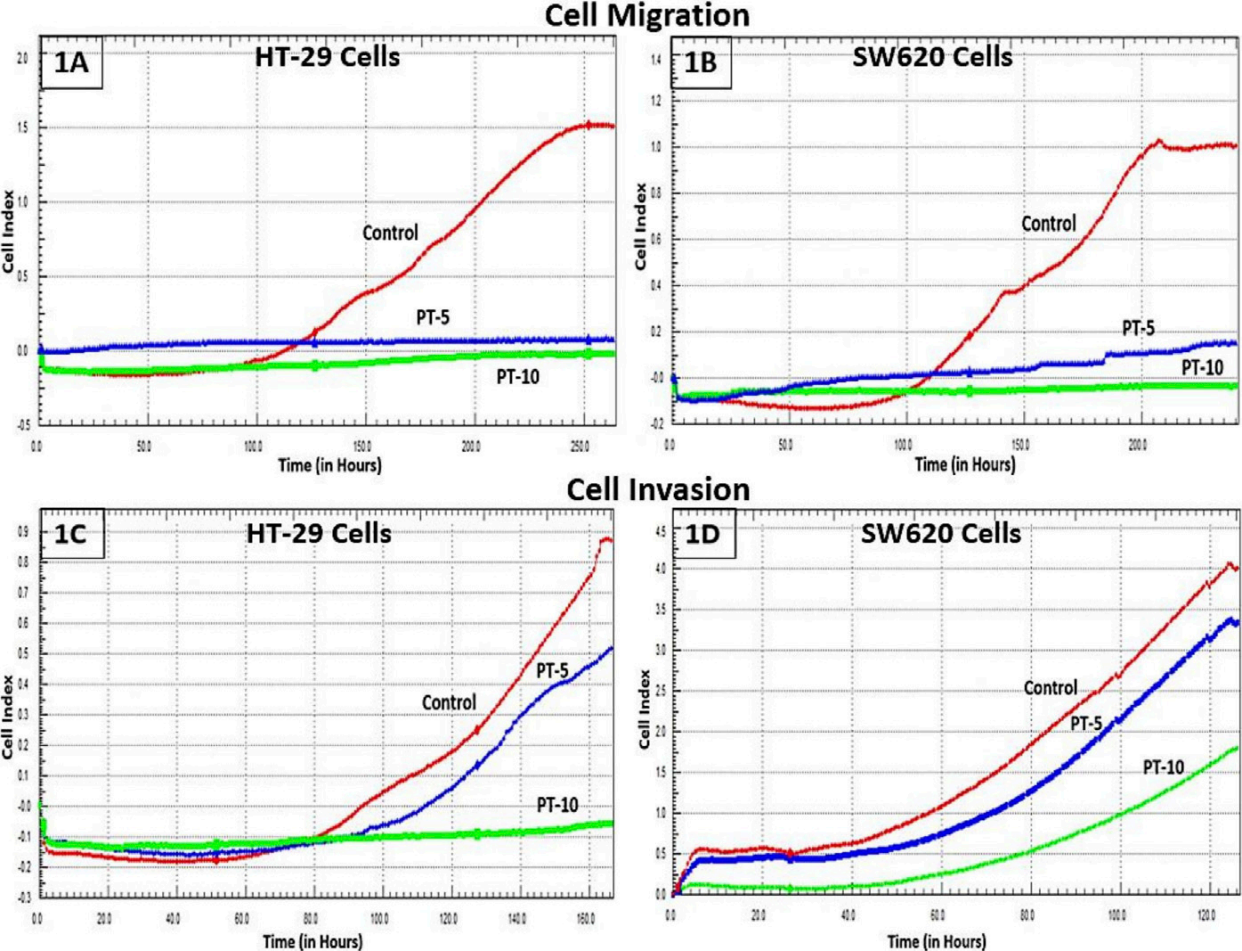
Effect of the PT complex on migration and invasion in real-time. **(A)** HT29 cells. **(B)** SW620 cells. Using the xCELLigence RTCA-DP system, the real-time migrations of cells were monitored. The invasion of **(C)** HT29 and **(D)** SW620 cells in Matrigel-coated upper chambers was monitored in real-time.

The motility and invasive capacity of cancer cells are the key factors for metastasis. HT-29 and SW62 cells exposed to different PT complex concentrations were shown to have decreased migration compared with controls. PT-complex-mediated inhibition of cell migration was time-dependent (*p* < 0.001, Figures 1A, B). Moreover, the reduction in cell migration is varied and depends on the doses of PT complex. Cells treated with the higher dose of PT complex (10 µM) showed a lower ability to migrate than those treated with a lower dose (5 µM). Based on the results of this study, the PT complex inhibits the migration of CRC cells in a dose-dependent manner. Additionally, the PT complex shows dose-dependent inhibition in the cell invasion capacity of HT-29 and SW620 cells (Figures 1C,D).

PT-complex-mediated inhibition of cell migration was further confirmed by an *in vitro* wound healing assay. This assay is a simple method for studying the directional *in vitro* cell migration, mimicking *in vivo* wound healing through cell migration.

**FIGURE 2:**
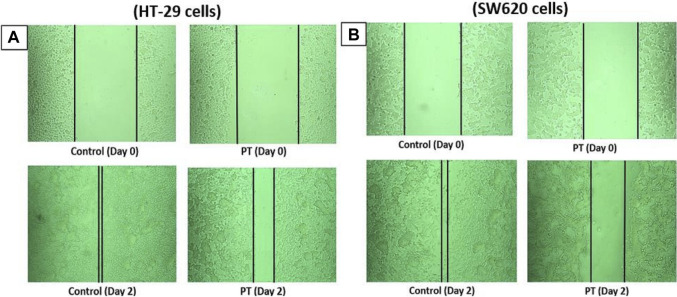
Effect of the PT complex on wound healing. In a six-well plate, **(A)** HT29 and **(B)** SW620 cells were seeded in complete medium and allowed to grow to 90% confluency. Monolayers were scratched with tips and then washed with PBS. The medium was replaced with fresh medium supplemented with DMSO or PT complex and kept in the incubator for 48 h. The cells were viewed by microscopy and digital images were taken. Each experiment was repeated three times, and images were taken to confirm the reproducibility of the result.

The wound healing assay was evaluated by monitoring the reduction and closing of wounded areas formed by scratches on a cell monolayer. After 48 h of incubation, the results show that compared with control cells, the gap distances in PT-complex-treated SW620 and HT-29 cells were larger (Figures 2A,B). The gap size in SW620 and HT-29 cells treated with the PT complex was almost three times greater than the untreated controls, indicating that the PT complex significantly suppressed the migration of both cancer cell lines.

### Effect of the PT complex on the expression of EMT markers in CRC cells

**FIGURE 3:**
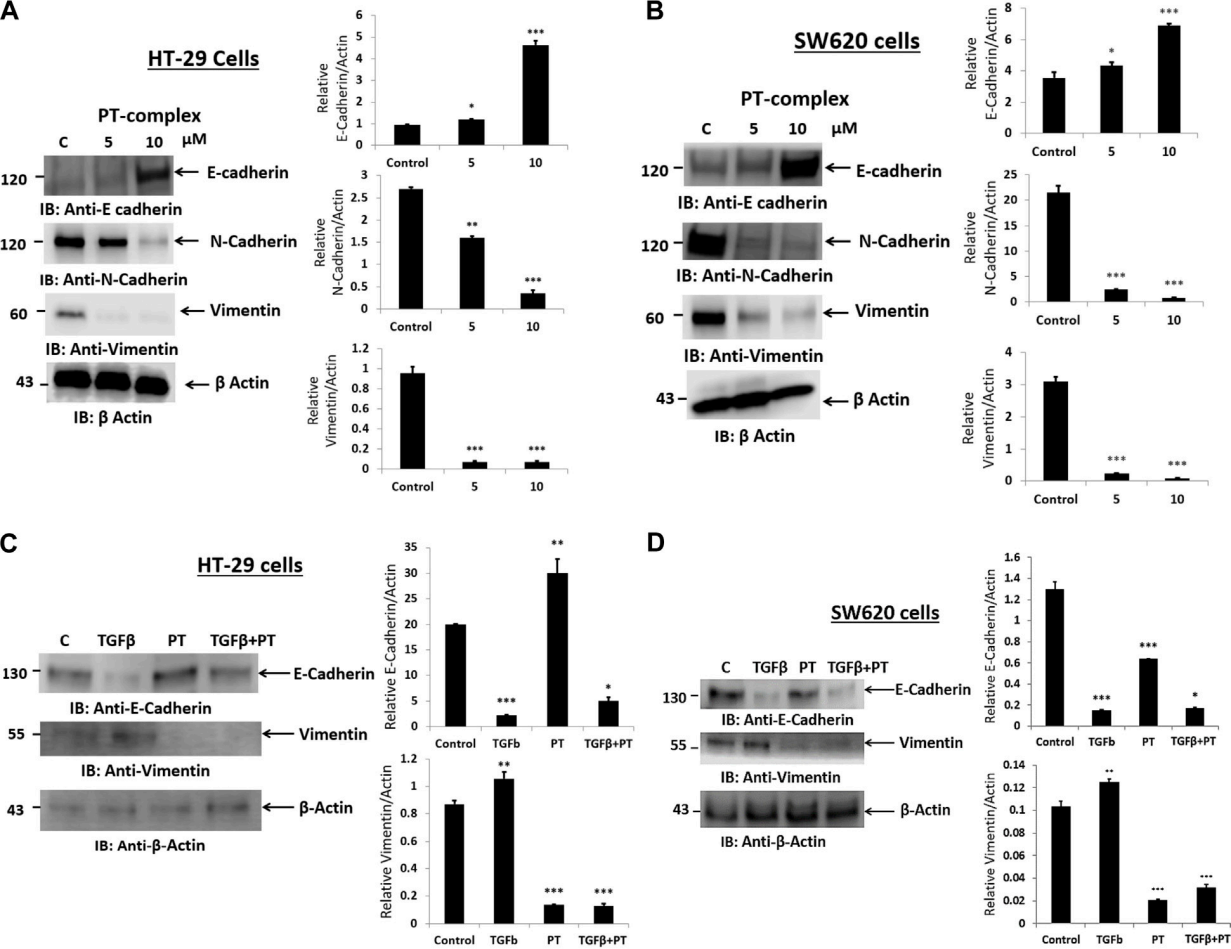
Effect of PT complex (PT) and TGF-β on EMT markers. **(A)** HT-29 and **(B)** SW620 cells were treated with different concentrations of PT complex for 24 h at 37°C. **(C)** HT-29 and **(D)** SW620 cells were treated with TGF-β (10 ng/m) in the presence or absence of PT complex. Total cell lysates were immunoblotted with the indicated antibodies. Densitometric analysis was conducted. The intensity of the protein bands was semi-quantified relative to β-actin, which was used as an internal control, and plotted as relative protein expression to the untreated control. The bar graphs are presented as the mean ± SD of three independent experiments. *p < 0.05, **p < 0.01 ***p < 0.001 vs. control.

Alteration of E-cadherin and N-cadherin can promote cancer metastasis through cell migration and invasion. Therefore, to study the impact of the PT complex on EMT markers in both cancer cell lines (HT29 and SW620), Western blotting was performed to assess the protein expression in the total lysates of untreated control and PT-complex-treated (5µM and 10 µM) cells. The results showed that cells treated with PT complex revealed a significant reduction in mesenchymal markers. Protein expression of N-cadherin and vimentin with a clear enhanced expression of epithelial marker protein E-cadherin, in comparison to control (Figures 3A, B). In contrast to control cells, PT-complex-treated cells showed significant concentration-dependent prevention in altering EMT markers. The higher dose of PT complex (10 µM) resulted in a higher increase in the expression of E-cadherin and the lowest expression of vimentin and N-cadherin, which confirmed the capability of the PT complex to reverse the EMT in HT-29 and SW620 cell lines (Figures 3A, B).

### PT complex inhibits TGF-β-induced EMT markers

PT-complex-treated (10 µM) HT-29 and SW620 cells showed significant upregulation of epithelial marker protein and downregulation of mesenchymal marker proteins. As TGF-β is a well-known inducer of the EMT process during migration and metastasis, the role of the PT complex in TGF-β-induced alteration in the EMT markers in HT-29 and SW620 cell lines was elucidated. The treatment with PT complex alone led to an increase in the protein expression of the epithelial marker E-cadherin and a decrease in the protein expression of the mesenchymal marker vimentin. In the absence of the PT complex, cells with TGF-β (10 ng/mL) reduced the protein expression of E-cadherin and elevated the protein expression of vimentin in both cells, thus confirming the induction of the EMT process. However, treatment of the cells with TGF-β in the presence of the PT complex showed opposite results: the PT complex increased E-cadherin expression whereas vimentin expression was suppressed relative to the group treated with TGF-β alone (Figures 3C,D). Thus, the PT complex was found to block the TGF-β-mediated EMT in the cancer cell. Moreover, our results showed that the inhibitory effect of the PT complex on TGF-β-induced altered EMT was greater in HT29 cells than in SW620 cells. (Figures 3C,D).

### PT complex inhibits TGF-β-induced phosphorylation of SMAD3

**FIGURE 4:**
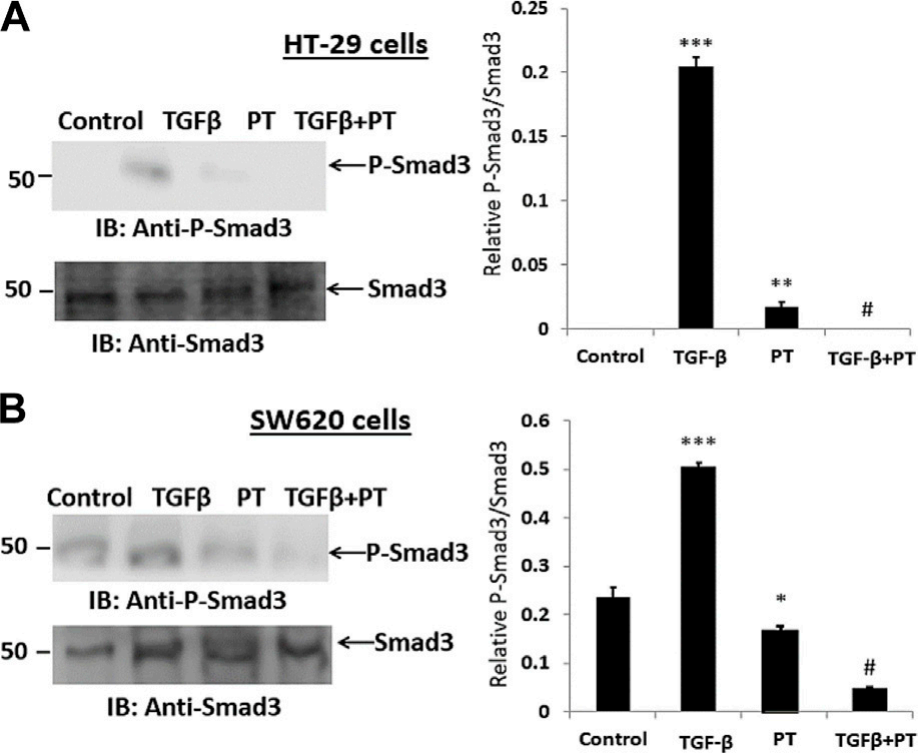
The PT-complex (PT) inhibits TGF-β-induced activation of SMAD3. **(A)** HT-29 cells and **(B)** SW620 cells were treated with TGF-β (10 ng/m) in the presence or absence of PT complex. Total cell lysates were immunoblotted with the indicated antibodies. Densitometric analysis was conducted as follows: protein band intensity was semi-quantified relative to β-actin, which was used as an internal control, and plotted as relative protein expression to the untreated control. The bar graphs are presented as the mean ± SD of three independent experiments. *p < 0.05, ***p < 0.001 vs. control; ***p < 0.001 vs. TGF-β.

Next, we examined the effect of the PT complex on the TGF-β-SMAD pathway as activated SMAD3 is a critical effector in TGF-β signaling and has a significant role in transcriptional regulatory responses that favor metastasis. We observed that the PT complex effectively suppressed the activation/phosphorylation of SMAD3 in SW620 cells in cells exposed to the PT complex only, whereas TGF-β in the absence of the PT complex significantly upregulated phosphorylated (P)-SMAD3 expression. Furthermore, in the presence of the PT complex, the TGF-β-induced phosphorylation of SMAD3 was markedly suppressed in HT-29 and SW620 cells (Figures 4A,B). Thus, the findings suggest that the PT complex inhibits the TGF-β-SMAD3 pathway and may have a therapeutic potential in inhibiting migration and invasion in CRC metastasis.

### PT complex inhibits novel invasion-migration-related genes in CRC

Global gene expression of untreated and PT-complex-treated SW620 cells revealed many novel downregulated and upregulated genes from microarray data (Supplementary Table 1 and 2) *MAP4*, *STAT3*, *KLC2*, *ZDHHC14*, *POSTN*, *WNT10B*, *PIEZO2*, *FXYD*, *IGSF6*, and *MTCH2* genes are involved in invasion-migration processes. These genes were significantly downregulated in PT-complex-treated cells. Microtubule-associated protein 4 (MAP4), a key regulator of invasion and migration, was considerably downregulated in SW620 cells. STAT3, which promotes tumor growth and metastasis, was also significantly downregulated in PT-complex-treated cells. The PT complex downregulated the gene expression of kinesin-1 light chain 2 (*KLC2*), mechanosensing *PIEZO2*, mitochondrial carrier homolog 2 (*MTCH2*), and *ZDHHC14*, which are known to enhance the invasion and migration of cancer cells. *POSTN* (periostin), known to be secreted from cancer-associated fibroblast (CAF), and wingless-type MMTV integration site family member 10B (*WNT10B*), which promote EMT, were also found to be inhibited by the PT complex. Cadherin 20 (CDH20) is a type II classical cadherin linked with cell-to-cell adhesion. In SW620 cells, upregulation of the gene expression of the proteins known to inversely regulate cell migration and invasion was observed. Moreover, the PT complex upregulated the expression of specific potential cancer suppressor genes, such as cyclin G2, V-set and transmembrane domain containing 4 (*VSTM4*), coiled-coil domain containing 149 (*CCDC149*), CD2 molecule, and cyclin G2.

### Dysregulation of signaling pathways by PT-complex

**FIGURE 5:**
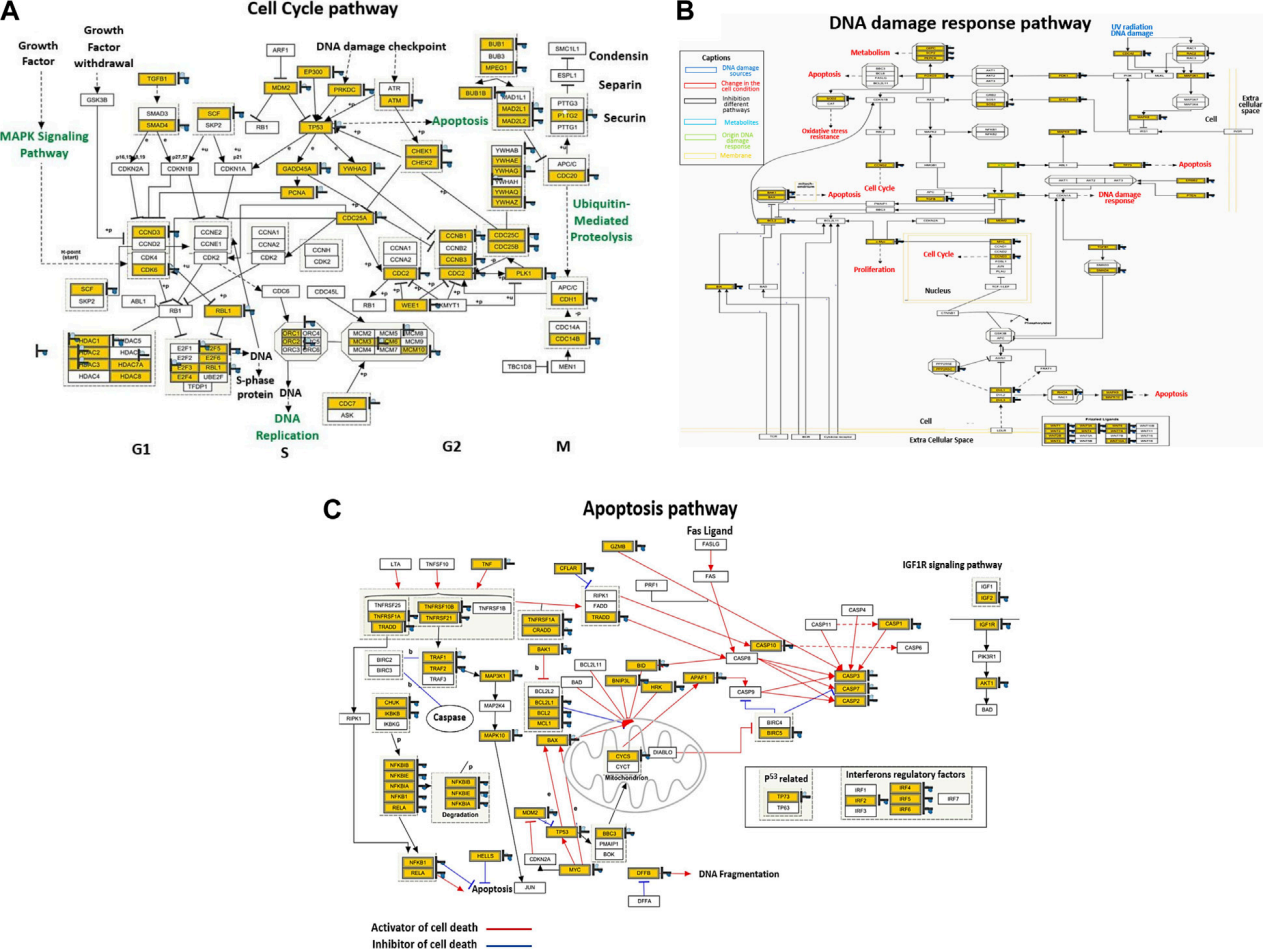
The PT complex regulates cellular pathways. **(A–C)** Illustration of the cell cycle, DNA damage response, and apoptosis pathways. Immunoblotting of total cell lysates was performed with the indicated antibodies.

Global mRNA expression profiling was performed on control and PT-complex-treated cells to determine the cellular pathways regulated by the drug. Pathway analysis of differentially expressed genes in PT-treated cells showed enhancement in genes related to cell cycle, apoptosis, and DNA damage response pathways. In PT-complex-treated cells, analysis of the cell cycle pathway revealed downregulation of the *TGF-β1* and *SMAD4* genes, confirming our finding that the PT complex inhibits TGF-β-SMAD signaling (Figure 5A). While observing the DNA damage response pathway, PT inhibited Rac and CDC42 (Figure 5B). The Rho family, including the GTPases Rho, Rac, and CDC42, are key players in cell growth, invasion-migration, polarity, adhesion, and cancer metastasis. The apoptosis pathway showed inhibition of BCL2L1, BCL2, and MCL1 and induction of p53, Bax, Caspase-3, and Caspase-7 (Figure 5C), confirming our previous finding (Al-Khayal et al., 2020).

## Discussion

Cancer cell resistance to the front-line chemo-drugs has been recognized as a significant impediment in CRC treatment (Wang et al., 2019). Effective treatments for the metastasis of CRC are limited. Hence, the identification of safer and more effective drugs for CRC treatment is crucial. Our previous studies reported that the PT complex possesses potent anticancer activity in HT-29 and SW620 cell lines. It was demonstrated that the PT complex markedly suppresses cell viability by inducing oxidative stress and enhancing apoptosis in CRC cells *in vitro.*

Migration and invasion of the cancer cells are linked to an advanced stage of malignancy and related to almost 90% of cancer-associated deaths (Guan, 2015). A major characteristic of metastasis is the invasive capability of the cancer cells, primarily driven by cell migration (Wu et al., 2020; Huang et al., 2022). Thus, the current study investigated whether PT complex treatment can inhibit cancer cell migration and the invasion of HT-29 and SW620 cells. Our findings revealed that the PT complex induced a significant dose-dependent inhibition of cell invasion and migration in CRC cell lines. This indicates that the PT complex has effective anti-migration and invasion activities and thus can prevent cancer metastasis in CRC.

Epithelial-to-mesenchymal transition (EMT) is considered a crucial multistep physiological and morphological process in normal cells, but it also triggers cancer invasion and migration leading to metastasis (Wang et al., 2020). The alteration in the expression of three major EMT biomarkers, such as E-cadherin, vimentin, and N-cadherin, may lead to decreased cell adhesion, the enhancement of mesenchymal markers, and the suppression of epithelial markers (Pavlič et al., 2021). Therefore, it is crucial to monitor the impact of the drug on the expression of the EMT markers, as their alteration increases the invasion and migration capacity of CRC and is linked to disease progression. The effect of different concentrations of PT complex on the protein expression of EMT markers in human HT- 29 and SW620 cells showed a concentration-dependent downregulation of EMT-inducing factors such as N-cadherin and vimentin, and upregulation of the EMT-suppressing factor E-cadherin in the cancer cell lines indicates that the drug induces its anti-migration and invasive activity by modulating the cancer-induced EMT switch in CRC cells. Dose-dependent drug regulation reveals that knowledge of the appropriate therapeutically effective drug concentrations is essential in cancer treatment (Senapati et al., 2018).

It is well known that CRC represents a group of heterogeneous molecular diseases characterized by various genetic and epigenetic changes (Hong, 2018). Many signaling pathways regulate the EMT process and cause tumor progression (Cao et al., 2015; Vu and Datta, 2017; Deshmukh et al., 2021). However, TGF-β is the key promoter of the EMT, and in the tumor microenvironment, it enhances CRC progression and metastasis (Lu et al., 2020). In the early phase of oncogenesis, TGF-β can induce cell apoptosis. However, in the advanced stage, it promotes tumorigenesis by inducing EMT progression. The TGF-β1-SMAD signaling pathway has been reported to be a dominant mechanism for promoting the EMT of cancer cells (Principe et al., 2014). Thus, this study also examined whether PT can inhibit TGF-β-induced EMT and SMAD3 expression in CRC cell lines. The results demonstrated that TGF-β treatment in the absence of the PTcomplex downregulated E-cadherin expression and upregulated vimentin expression relative to the untreated control cells. By contrast, the PT complex significantly restored the altered expression of EMT markers in TGF-β-treated cells. Furthermore, the results showed that TGF-β increased the expression of phosphorylated SMAD3 compared with the untreated CRC cells. PT complex treatment significantly decreased the expression of TGF-β-induced phosphorylated SMAD3 in both cell lines. Several studies have shown the role of SMADs in TGF-β-induced EMT (Avila-Carrasco et al., 2019; Hua et al., 2020). Thus, the PT-complex-mediated suppression of the SMAD activation may have suppressed the TGF-β1-SMAD pathway, which may have led to the restoration of the EMT and attenuation of invasiveness and metastasis in HT-29 and SW620 cells. Previous studies have reported that the PT complex is an effective anticancer drug (Azam et al., 2017; Al-Khayal et al., 2020). However, this study is the first to show that the PT complex significantly inhibits the invasion and migration of CRC cells through the attenuation of TGF-β-induced activation of the TGF-β-SMAD pathway and EMT process.

Microarray data revealed the downregulation of many novel genes related to invasion and migration. In gastrointestinal cancer, particularly esophageal squamous cell carcinoma, MAP4 has been reported as a therapeutic target and the key regulator of cell invasion and migration. Moreover, the expression of MAP4 has been observed in breast cancer cell lines (Jiang et al., 2016). Additionally, STAT3 was found to be significantly downregulated in PT-complex-treated cells. IL6-STAT3 signaling is well known to play a critical role in colorectal cancer metastasis (Gargalionis et al., 2021). The PT complex suppressed kinesin-1 light chain 2 (KLC2), which enhances the invasion and migration of non-small cell lung cancer cells (Wang et al., 2015) (32). ZDHHC14 overexpression promotes the invasion and migration of scirrhous-type gastric cancer (Oo et al., 2014). POSTN (Periostin) secretion from cancer-associated fibroblast (CAF) promotes cancer stemness in head and neck cancer (Yu et al., 2018). WNT10B is known to regulate gastric cancer progression by promoting EMT (Pardo-Pastor et al., 2018). The mechanosensing PIEZO2-initiated signaling pathway has associations with different characteristics of cancer invasion and metastasis (Wu et al., 2017). MTCH2 (mitochondrial carrier homolog 2) has been reported to play a critical role in tumor invasion in malignant glioma (Yuan et al., 2021). Pathway evaluation of differentially expressed genes in PT-complex-treated cells revealed enhancement in genes related to cell cycle, apoptosis, and DNA damage response pathways.

Authenticated cancer cell lines retain most of the genetic properties of the original cancer when maintained in the right conditions with appropriate controls. However, it is crucial to consider that cell lines have many limitations that might not reflect the factors that influence the action of a drug *in vivo*. Hence, further drug testing *in vivo* is recommended to address the shortcomings of *in vitro* studies.

In conclusion, our investigations demonstrate that the PT complex can successfully inhibit cancer metastasis by impairing the key pathway responsible for cellular migration and invasion in CRC. Cell invasion and metastasis are the leading cause of cancer-related deaths in the advanced stages of the disease. Therefore, the anti-migratory property of the PT complex can make it a potent compound that improves the effectiveness of chemotherapy and, thus, may improve the survival rate in cancer patients.

## Data Availability

The original contributions presented in the study are included in the article/supplementary material, further inquiries can be directed to the corresponding authors.
