# Supplementary material for: The platinum coordination complex inhibits cell invasion-migration and epithelial-to-mesenchymal transition by altering the TGF-β-SMAD pathway in colorectal cancer
Source: Front Pharmacol. 2023 Nov 8;14:1178190. doi: 10.3389/fphar.2023.1178190 (PMC10679924; doi:10.3389/fphar.2023.1178190)
Supplement: Supplementary file 1 [file DataSheet1.docx]

Supplementary Table 1: PT-mediated downregulated genes

| **GeneSymbol** | **GeneName** | **Log FC (Con vs PT)** |
| --- | --- | --- |
| MAP4 | microtubule-associated protein 4 | -7.53586 |
| ORM2 | orosomucoid 2 | -7.526372 |
| TMEM184C | transmembrane protein 184C | -7.388319 |
| STAT3 | signal transducer and activator of transcription 3 (acute-phase response factor) | -7.3260384 |
| C11orf54 | chromosome 11 open reading frame 54 | -7.3070145 |
| PTGER4P2-CDK2AP2P2 | PTGER4P2-CDK2AP2P2 readthrough transcribed pseudogene | -7.2603226 |
| KLC2 | kinesin light chain 2 | -7.236768 |
| ZDHHC24 | zinc finger, DHHC-type containing 24 | -7.1688843 |
| POSTN | periostin, osteoblast specific factor | -7.1645546 |
| WNT10B | wingless-type MMTV integration site family, member 10B | -6.9836893 |
| JPH2 | junctophilin 2 | -6.9534245 |
| COL17A1 | collagen, type XVII, alpha 1 | -6.8679457 |
| RARRES2 | retinoic acid receptor responder (tazarotene induced) 2 | -6.7449465 |
| SNORD76 | small nucleolar RNA, C/D box 76 | -6.682482 |
| PIEZO2 | piezo-type mechanosensitive ion channel component 2 | -6.6658792 |
| FXYD3 | FXYD domain containing ion transport regulator 3 | -6.6557026 |
| FOXN1 | forkhead box N1 | -6.6417003 |
| IGSF6 | immunoglobulin superfamily, member 6 | -6.330265 |
| MID1 | midline 1 | -6.3122263 |
| CACNA1E | calcium channel, voltage-dependent, R type, alpha 1E subunit | -6.311795 |
| RBM44 | RNA binding motif protein 44 | -6.302739 |
| FAM49B | family with sequence similarity 49, member B | -6.2199574 |
| CCNG2 | cyclin G2 | -6.0834217 |
| MTCH2 | mitochondrial carrier 2 | -6.0099 |
| ATG2A | autophagy related 2A | -5.9656467 |
| NXNL1 | nucleoredoxin-like 1 | -5.923728 |
| CNTRL | centriolin | -5.9148617 |
| FAM207A | family with sequence similarity 207, member A | -5.9107647 |
| UBASH3B | ubiquitin associated and SH3 domain containing B | -5.6763926 |
| CD200 | CD200 molecule | -5.65549 |
| SMC1A | structural maintenance of chromosomes 1A | -5.6552696 |
| LINC00307 | long intergenic non-protein coding RNA 307 | -5.655258 |
| PKP3 | plakophilin 3 | -5.6509466 |
| PCOLCE | procollagen C-endopeptidase enhancer | -5.530995 |
| MAPKAP1 | mitogen-activated protein kinase associated protein 1 | -5.526904 |
| BAD | BCL2-associated agonist of cell death | -5.5124006 |
| MPST | mercaptopyruvate sulfurtransferase | -5.4969044 |
| LHPP | phospholysine phosphohistidine inorganic pyrophosphate phosphatase | -5.4668517 |
| RBM12B | RNA binding motif protein 12B | -5.432951 |

**Supplementary Table 2: PT-mediated upregulated genes**

| **GeneSymbol** | **GeneName** | **Log FC (Con vs PT)** |
| --- | --- | --- |
| SLC22A1 | solute carrier family 22 (organic cation transporter), member 1 | 13.422233 |
| DEF6 | differentially expressed in FDCP 6 homolog (mouse) | 10.973113 |
| VSTM4 | V-set and transmembrane domain containing 4 | 10.42411 |
| CD2 | CD2 molecule | 10.418517 |
| UBQLN3 | ubiquilin 3 | 10.103601 |
| CRMP1 | collapsin response mediator protein 1 | 10.027926 |
| PWWP2A | PWWP domain containing 2A | 9.896734 |
| PFDN2 | prefoldin subunit 2 | 9.881932 |
| POU2F2 | POU class 2 homeobox 2 | 9.76372 |
| TAF8 | TAF8 RNA polymerase II, TATA box binding protein (TBP)-associated factor, 43kDa | 9.744096 |
| KIF20B | kinesin family member 20B | 9.459025 |
| UBE2Z | ubiquitin-conjugating enzyme E2Z | 9.36309 |
| IPO5 | importin 5 | 9.127175 |
| PROKR1 | prokineticin receptor 1 | 8.799857 |
| CCDC149 | coiled-coil domain containing 149 | 8.775337 |
| CNTNAP5 | contactin associated protein-like 5 | 8.769547 |
| FLJ40536 | FLJ40536 protein | 8.669573 |
| PTRF | polymerase I and transcript release factor | 8.590061 |
| TAAR3 | trace amine associated receptor 3 (gene/pseudogene) | 8.589442 |
| EVI5L | ecotropic viral integration site 5-like | 8.57083 |
| USP22 | ubiquitin specific peptidase 22 | 8.564262 |
| RAP1GAP | RAP1 GTPase activating protein | 8.559361 |
| RIBC1 | RIB43A domain with coiled-coils 1 | 8.395233 |
| COL8A2 | collagen, type VIII, alpha 2 | 8.384612 |
| FGF4 | fibroblast growth factor 4 | 8.364143 |
| TMEM130 | transmembrane protein 130 | 8.343352 |
| ZNF561 | zinc finger protein 561 | 8.343205 |
| CEP295 | centrosomal protein 295kDa | 8.338645 |
| ACTN2 | actinin, alpha 2 | 8.334025 |
| LYZL1 | lysozyme-like 1 | 8.333395 |
| CD163L1 | CD163 molecule-like 1 | 8.332844 |
| ZNRF3 | zinc and ring finger 3 | 8.330948 |
| RICTOR | RPTOR independent companion of MTOR, complex 2 | 8.258091 |
| LMNTD1 | lamin tail domain containing 1 | 8.24646 |
| PTF1A | pancreas specific transcription factor, 1a | 8.241149 |
| SELK | selenoprotein K | 8.198766 |
| CDH20 | cadherin 20, type 2 | 8.198692 |
| MAP7 | microtubule-associated protein 7 | 8.190267 |
| PRR34-AS1 | PRR34 antisense RNA 1 | 8.184764 |
| NAP1L5 | nucleosome assembly protein 1-like 5 | 8.173952 |
| NES | nestin | 8.158358 |
